# Uncovering the correlations between the structure and valence tautomerism in Co(Dioxolene)2Py2 crystals.

**DOI:** 10.1063/4.0000919

**Published:** 2025-10-27

**Authors:** Marcelo F. F. Alecrim, Carlos B. Pinheiro, Simone S. Alexandre

**Affiliations:** Universidade Federal de Minas Gerais (UFMG), Belo Horizonte, Minas Gerais, Brazil

## Abstract

Valence tautomers are electronically labile compounds that can switch between two distinct energy states with two different electronic distributions, which modifies the physical properties of the compound. This interconversion is triggered by external stimuli, such as temperature increase, pressure, application of an electric field, light incidence, and others [1,2]. The present study aims to investigate valence tautomerism (VT) characteristics in trans pyridine-solvated Co(dioxolene)_2_(Py)_2_ complexes, where dioxolene represents 3,5-di-tert-butyl semiquinone and 3,5- di-tert-butyl catecholate, and Py stands for pyridine. Each dioxolene form is associated with a specific energy state: 3,5-di-tert-butyl catecholate corresponds to the low-spin (LS) state, while 3,5-di-tert-butyl semiquinone is in the high-spin (HS) state, both of which are stable in given temperatures. In this compound, the interconversion is directly linked to a change in cobalt's oxidation state from LS-Co(III) to HS-Co(II), allowing the spin state to be identified via single-crystal X-ray diffraction (SCXRD) by analyzing cobalt's bond lengths. However, the chemical environment appears to be crucial for the occurrence of valence tautomerism in solvated Co(dioxolene)_2_(Py)_2_ crystals [3]. Different complex/solvent crystal ratios (1:0, 2:1, and 1:2) were analyzed using SCXRD across a wide temperature range (100-300 K) to assess the influence of crystal packing and solvation on VT interconversion. Among these, only the 2:1 complex/solvent ratio (Co(dioxolene)_2_(Py)_2_⋅0.5Py) exhibited VT , while the other two remained in the low-spin state throughout the entire investigated temperature range.

Our findings demonstrate that the torsion angle (θ in Figure 1) between pyridine planes and dioxolene centroids in Co(dioxolene)_2_(Py)_2_⋅0.5Py crystal directly correlates with the LS-Co(III) ⇔ HS-Co(II) VT interconversion. The HS-Co(II) state can be observed when θ exceeds ∼17.5°. A search of the CCDC database revealed that this pattern extends to all compounds structurally similar to Co(dioxolene)_2_(Py)_2_, suggesting a direct correlation between molecular torsion and spin state. In the 2:1 complex/solvent ratio, only half of the molecules in the unit cell undergo the phase transition. This partial conversion appears to result from π-stacking interactions between the solvent and pyridine ligands, which restrict pyridine rotation and consequently inhibit the transition.

To characterize the valence tautomerism properties of these compounds, we also performed DFT calculations using the structures obtained from SCXRD measurements at different temperatures as starting points using the SIESTA software package (version 4.1.5) [4]. Electronic structure calculations have become increasingly important for characterizing and even predicting the occurrence of valence tautomerism [5]. The experimental magnetic susceptibility curve of Co(dioxolene)_2_(Py)_2_⋅0.5Py served as a reference to validate and select the appropriate functional for the calculations. Among the tested functionals, the Local Density Approximation (LDA) [6] functional proved most suitable, as it generally reproduced the main features of the experimental magnetic susceptibility curve. After selecting the functional, we performed atomic position optimizations for different temperature values. For starting points below the transition temperature, we observed a significant reduction in both bond lengths and torsion angles, with both parameters converging to values characteristic of the LS state. In contrast, calculations using starting points above the transition temperature maintained bond lengths consistent with the HS state, while the pyridine plane torsion angles remained above the critical value (∼17.5°). These results reinforce the existence of a correlation between torsion of Pyridine’s plans and spin state of these molecules and suggests a need of a closer look at the role of the so called innocent ligands in the VT observation
